# The recovery of the microbial community after plaque removal depends on periodontal health status

**DOI:** 10.1038/s41522-023-00441-0

**Published:** 2023-10-07

**Authors:** Xiaoqing Li, Cheng Yu, Bing Zhang, Xiaogang Shan, Wenjun Mao, Zicheng Zhang, Chunyan Wang, Xiaoxia Jin, Jinfeng Wang, Hui Zhao

**Affiliations:** 1 The Third Clinical Institute Affiliated to Wenzhou Medical University/Wenzhou People’s Hospital/Wenzhou Maternal and Child Health Care Hospital/The Third Affiliated Hospital of Shanghai University, Wenzhou, Zhejiang China; 2 Jiangyin Stomatological Hospital/Jiangyin Oral Disease Preventive Treatment, Jiangyin, Jiangsu China; 3 University of Chinese Academy of Sciences, Beijing, China; 4 Beijing Institutes of Life Science, Chinese Academy of Sciences, Beijing, China; 5 School of Biomedical Engineering, Hainan University, Haikou, Hainan China; 6 Henan Key Laboratory of Industrial Microbial Resources and Fermentation Technology, Nanyang Institute of Technology, Nanyang, Henan China; 7 College of Food Science & Nutritional Engineering, China Agricultural University, Beijing, China

**Keywords:** Plaque, Biofilms, Clinical microbiology

## Abstract

Plaque accumulation and microbial community changes are important causes of periodontal disease. Cleaned plaque microorganisms will reattach to form biofilms, but the recovery and outcome of plaque microbial communities in different periodontal health states remain unknown. In this study, we tracked the biofilm remodeling process in 206 dental plaque samples from 40 healthy periodontal, gingivitis and periodontitis volunteers at 6 time points before and after supragingival scaling. We found that microbial communities of different periodontal states changed asynchronously during the process, and the more severe the periodontal disease condition, the more lagged the recovery of plaque microorganisms to their original state after cleaning; this reflected a higher degree of plaque development in periodontitis samples. The plaque index and bleeding index were significantly correlated with plaque recovery, especially the recovery of bacteria such as *Abiotrophia* and *Capnocytophaga*. Meanwhile, we found that the microbial community structure of different periodontal health states was most similar at the Day 3 after plaque cleaning, and the communities gradually differentiated and developed in different directions. *Abiotrophia* and other bacteria might play an important role in determining the development trend of plaque biofilms. The discovery of specific time points and bacteria was of great value in clarifying the pathogenesis of periodontal disease and in seeking targets for prevention and treatment.

## Introduction

Periodontal diseases, including gingivitis and periodontitis, are the most common oral diseases that cause tooth loss in adults. Periodontitis is considered a continuous process of health deterioration in gingivitis. Imbalanced in plaque microorganisms cause gingival inflammation in the early stages, and long-standing gingivitis develops into chronic periodontitis^1^. It was estimated that periodontitis affects more than 50% of the world’s population, with a global prevalence of severe periodontitis of approximately 11.2% from 1990 to 2010^2^. According to the Global Burden of Disease Study (2016), severe periodontal disease is a major epidemic disease worldwide^3^. The global burden of periodontal disease increased by 57.3% between 1990 and 2010^4^, and the global productivity loss due to severe periodontal disease was estimated at $54 billion per year in 2010^5^. Many systemic diseases, such as diabetes, cancer, and Alzheimer’s disease, as well as premature birth, are associated with the periodontitis^6,7^.

Periodontal disease is diagnosed based on the presence and degree of periodontal inflammation, usually measured using probing bleeding, probing depth, clinical attachment loss, and radiographs to assess the type and extent of alveolar bone loss^8^. Probing depth is the distance from the gingival margin to the gingival sulcus or the bottom of the periodontal pocket. Clinical attachment loss is the distance from the enamel junction to the bottom of the gingival groove or periodontal pocket. The accuracy and repeatability of probing depth and clinical attachment loss measurements are important because the clinical diagnosis of periodontitis is mainly based on these two parameters. Different diagnostic criteria may lead to variations in the diagnosis of the extent of disease^9^. The Centers for Disease Control and Prevention/American Academy of Periodontology proposed a definition of periodontology in 2007^8^ and revised it slightly in 2012. According to this definition, periodontitis is considered to be present when there is ≥3 mm of clinical attachment loss in ≥2 adjacent sites and ≥4 mm of probing depth in ≥2 adjacent sites (not on the same tooth), or ≥5 mm of probing depth in one adjacent site^10^. This standard is used worldwide^11^. Additionally, indices such as bleeding on probing, gingival recession and plaque index are often used as indicators to assist in the diagnosis of periodontal disease.

Dental plaque microbial membranes growing on the tooth surface are the primary cause of periodontal disease^12^. Plaques are communities of microbes that adhere to the surface of teeth. Due to the strong specificity of bacterial adhesion, the attached bacteria remain adhered to the tooth surface despite saliva flow, tongue movement and the mechanical force of rinsing with water, the bacteria do not fall off easily^13^. The plaque of patients with chronic periodontitis has a unique flora composition, with bacteria such as *Porphyromonas gingivalis*, *Tannerella forsythia*, *Prevotella intermedia* and *Treponema denticola* widely recognized as periodontal pathogens^14^. Bacterial interactions and functional profiles in healthy and periodontitis groups were significantly different, and the complexity of community correlation networks increased during the development of gingivitis and decreased during the development of periodontitis. Taxon such as *Eubacterium nodatum*, *Eubacterium saphenum*, *Filifactor alocis* and *Fretibacterium fastidiosum* showed increased transcriptional activity compared to periodontal pathogens during disease progression. These taxa might be associated with the progression of periodontal disease, suggesting that the shift in microbiota from healthy to periodontitis was accompanied by changes in the structure and complexity of bacterial networks^15^.

Periodontitis is difficult to eradicate, while gingivitis and healthy conditions can be interchangeable. Plaque removal can effectively prevent the occurrence of gingivitis and reduce the progression to periodontitis. Regular cleaning of dental plaque by supragingival scaling is the most common method for the clinical prevention and treatment of periodontitis^16^. Supragingival ultrasonic scaling could change the microbiota to reduce bacterial load and dental calculus deposition on the tooth surface^17^. Longitudinal sampling and microbiome analysis during the phase of progressive microbial adhesion and plaque re-accumulation after supragingival scaling could effectively track the process of plaque biofilm formation. Previously, we compared the dynamics of saliva and plaque flora after plaque removal^18^, but this study did not address direct comparisons between different periodontal states during this process. It is important to understand the specific changes in microbiome composition associated with periodontal health status, particularly the microbiota associated with gingivitis that play a role in the onset of periodontitis.

In this study, we removed dental plaque by ultrasonic cleaning on volunteers with different periodontal states (periodontal health, gingivitis, and periodontitis) and collected supragingival plaque samples at different time points before and after scaling. Comparative analysis was performed after detection the samples by 16S rRNA gene sequencing to investigate the similarities and differences in plaque remodeling processes in different periodontal states after scaling. These findings will be helpful for developing microbial targeted therapy to prevent periodontitis and for guiding the prevention and treatment of chronic periodontitis.

## Results

### Baseline of initial plaque microorganisms in different periodontal health conditions

**Fig. 1:**
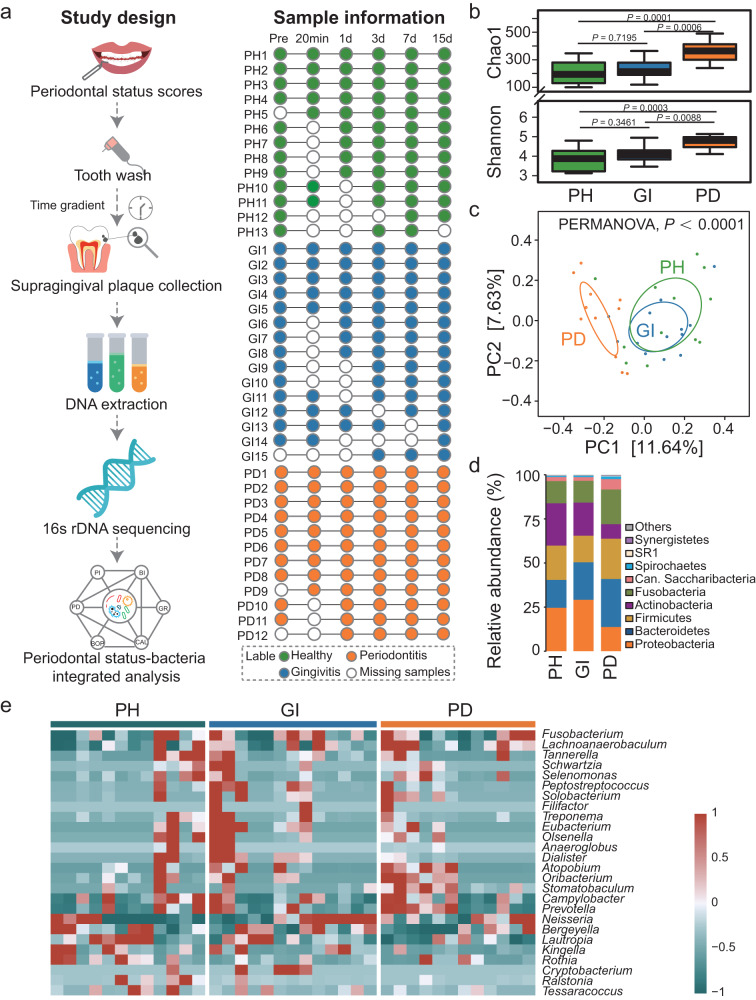
Cohort overview and changes in different periodontal health states. **a** Experimental flow chart and sample information. All volunteers with different periodontal health status were scaled, and supragingival plaque samples were collected at 6 time points before scaling and at 20 min, Days 1, 3, 7, and 15 after scaling. The V3-V4 region of the 16S rRNA gene was sequenced in each sample. Then, periodontal status and bacteria were integrated analysis; **b** Chao1 and Shannon indices in different periodontal states. Box plots showed center line as median, box limits as upper and lower quartiles, whiskers as 1.5 × interquartile range; **c** Principal coordinates analysis results of different periodontal states at the ASVs level, using PERMANOVA test; **d** Distribution in different periodontal states at phylum level; **e** Distribution of different bacteria in different periodontal states at the genus level; Pre represents pre-scaling, PH represents healthy periodontitis, GI represents gingivitis, and PD represents periodontitis. Can. Saccharibacteria stands for Candidatus Saccharibacteria. P < 0.05 represents a significant difference.

We recruited a total of 40 volunteers with varying periodontal health statuses for this study, including 13 with periodontal health, 15 with gingivitis and 12 with periodontitis (Fig. 1a). The general clinical data of the volunteers were collected (Supplementary Table 1). All volunteers underwent supragingival scaling, and we collected supragingival plaque samples at 6 time points before scaling and at 20 min, Days 1, 3, 7, and 15 after scaling. The V3-V4 region of the 16S rRNA gene was sequenced in each sample. Finally, 206 samples were successfully sequenced, yielding a total of 1,329,700 reads (2 × 250 bp), with an average of 64,549 reads per sample. After overlapping and merging into sequences, most of the sequence lengths were distributed between 400–430 bp (Supplementary Fig. 1a). Based on the results of the dilution curves, it was confirmed that the amount of sequencing data was sufficient and that the number of sequences accurately reflect the microbial diversity of each community (Supplementary Fig. 1b).

We analyzed initial plaque baseline microbes in healthy, gingivitis, and periodontitis volunteers prior to scaling and found that the Chao1 and Shannon indices were significantly higher in patients with periodontitis than in those with gingivitis and healthy individuals (*P* < 0.05); meanwhile, there was no significant difference between gingivitis patients and healthy individuals (Fig. 1b). Principal coordinates analysis based on the Bray‒Curtis (BC) distance demonstrated that plaque was able to form distinct clusters in volunteers with periodontitis compared to those with periodontal health and gingivitis (Fig. 1c, *P* < 0.0001, PERMANOVA test). Similar results were obtained in the DECODE analysis (Supplementary Fig. 2, *P* < 0.0001, PERMANOVA test). Further analysis revealed that the initial plaque microbes differed among different periodontal health conditions (Fig. 1d, e and Supplementary Fig. 3). The dental plaque of patients with periodontitis had more microbes, higher microbial community diversity, and a unique microbial community structure. Although the plaque of patients with gingivitis was more similar to healthy volunteers, there were differences in microbial composition.

### The more severe the degree of periodontal damage, the more lagging and switching the recovery of plaque flora

**Fig. 2:**
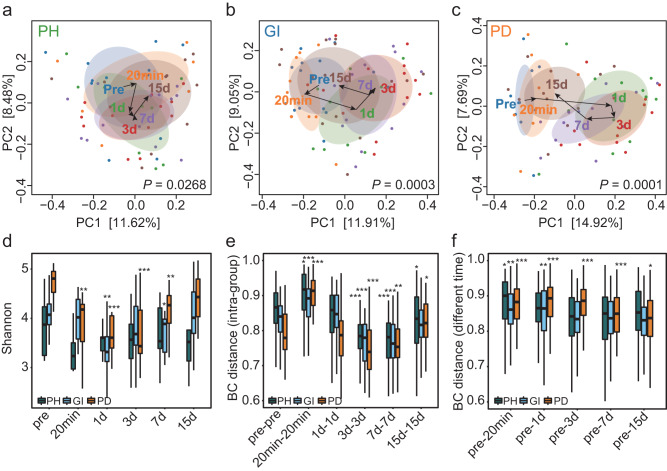
Changes in the microbial diversity of dental plaque under different periodontal health conditions after scaling. **a** Principal coordinates analysis for different time points before and after scaling in periodontal health samples at the ASVs level. **b** Principal coordinates analysis for different time points before and after scaling in gingivitis samples at the ASVs level. **c** Principal coordinates analysis for different time points before and after scaling in periodontitis samples at the ASVs level. Each point in the figure represents a sample, and each color represents a time point. The starting point of each arrow in the figure is the center of the circle for the group, and the direction of the arrow is a path, which is set according to the gradient direction of the continuous time after tooth cleaning (the order is pre-20 min-1d-3d-7d-15d). **d** Shannon indices of healthy periodontitis, gingivitis and periodontitis samples at different time points after scaling. The significant difference results were compared with the same periodontal health status before dental cleaning (pre). **e** Intragroup Bray‒Curtis distance of healthy periodontitis, gingivitis and periodontitis samples at the ASVs level. The significant difference results were compared with the same periodontal health status before dental cleaning (pre-pre). **f** Intergroup Bray‒Curtis distance of healthy periodontitis, gingivitis and periodontitis samples at the ASVs level. The significant difference results were compared with the same periodontal health status before dental cleaning. Box plots showed center line as median, box limits as upper and lower quartiles, whiskers as 1.5 × interquartile range. Pre represents pre-scaling, PH represents healthy periodontitis, GI represents gingivitis, and PD represents periodontitis. * represents *P* < 0.05, ** represents *P* < 0.01, *** represents *P* < 0.001.

To investigate the differences in the recovery process of plaque removal in different periodontal health conditions, we conducted longitudinal tracking of plaque flora at gradient time points before and after scaling in volunteers with periodontal health, gingivitis and periodontitis. Our analysis revealed that the amplitude of variation of dental plaque microorganisms in healthy periodontal volunteers changed significantly at different time points. (Fig. 2a, *P* = 0.0268, PERMANOVA test). After cleaning, plaque microorganisms changed dynamically with time and gradually recovered to their original state around Days 3–7. The structure of the supragingival plaque microbial community in patients with gingivitis (Fig. 2b, *P* = 0.0003, PERMANOVA test) and periodontitis (Fig. 2c, *P* = 0.0001, PERMANOVA test) also underwent similar transformation after cleaning. In particular, the independent clustering of periodontitis patients was more evident on Days 1, 3, and 7 after cleaning compared to the clustering before cleaning. We repeated the taxonomic assignment by employing the SILVA database and achieved consistent findings (Supplementary Fig. 4a–c). Our results suggested that the microorganisms associated with periodontal disease switched more than those associated with periodontal health. In addition, Similar results were obtained in the DECODE analysis (Supplementary Fig. 5a–c). After scaling, the quantity and diversity of dental plaque changed at different time points (Fig. 2d and Supplementary Fig. 6a–d). Patients with periodontal health and gingivitis reached the lowest point approximately 1 day after scaling and then began to reconstruct back to their initial state, while periodontitis plaques reached their lowest point approximately 3 days after scaling. The number of microorganisms in individuals with periodontal health began to recover earlier after dental cleaning, followed by those with gingivitis and periodontitis. Our findings suggested that the recovery of plaque flora was slower in individuals with more severe periodontal damage.

The BC distance within each different periodontal health states after scaling also switched with time, similar to α diversity (Fig. 2e). The BC distance reached its lowest point within the groups at approximately 3 days, and the changes in different periodontal states were synchronized. The intergroup BC distance between periodontal health and gingivitis plaque microorganisms at 3 days was most similar to the pre-cleaning distance (Fig. 2f), and periodontitis plaques were not completely synchronized with the changes in the other 2 states. Combined with the above results, these findings suggested that scaling played a role in early supragingival plaque formation; however, it could not inhibit the generation of plaques for an extended period of time. After deconstruction, the bacterial community began to regrow in approximately 1–3 days and gradually returned to its pre-cleaning state with increasing time. Compared to the remodeling process of periodontal health and gingivitis plaques, periodontitis plaques associated with poorer periodontal health had greater switching in supragingival plaque and their recovery was more delayed.

**Fig. 3:**
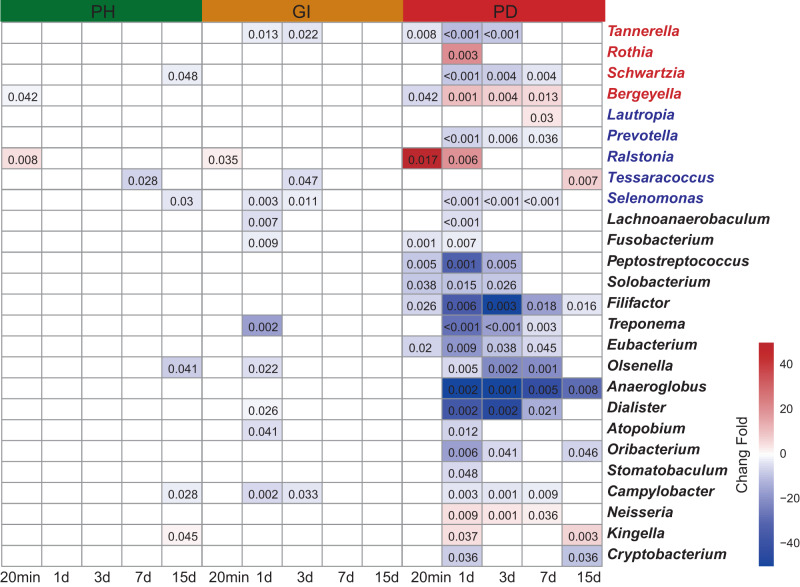
Changes in differential bacteria at different time points after scaling. Significantly different genera at each time point compared to before scaling at the genus level. Red shadows represent increased genera, while blue shadows represent decreased genera. The numbers in the box show *P*-*values* (Wilcoxon test). PH represents healthy periodontitis, GI represents gingivitis, and PD represents periodontitis. The red fonts represent the bacteria with significant differences after LEfSe analysis, ALDEx2, and ANCOM-BC analysis at the genus level. The blue fonts represent the bacteria with significant differences after any two of these analyses at the genus level.

Then, we explored the changes in bacteria before scaling (Fig. 1e and Supplementary Fig. 3) and at different time points after scaling (Fig. 3 and Supplementary Fig. 7) and found that with the deterioration of periodontal health, the switching in differential bacteria were more pronounced. The changes in bacteria were less in periodontal health plaques, followed by gingivitis plaques. Meanwhile, almost all of the bacteria changed in periodontitis plaques. The state of gingivitis plaques was in between healthy and periodontitis plaques. The changes in gingivitis plaques at Days 1 and 3 were more evident than those in healthy periodontitis plaques, but the changes were more evident at Days 1, 3, and 7 in periodontitis plaques, suggesting that the process of scaling and remodeling in the three periodontal states was different. The bacteria that changed significantly at Day 1 in gingivitis plaques also changed significantly in periodontitis plaques and showed a lag in recovery. Perhaps these bacteria played a role in the transition from gingivitis to periodontitis. We found that the recovery time of different bacteria after scaling was different in periodontitis plaques. After scaling, the bacteria that were reduced, such as *Fusobacterium*, returned to their initial level at Day 3, while *Tannerella* and other bacteria returned to their initial level at Day 7. However, *Schwartzia*, *Bergeyella*, and other bacteria did not recover until Day 15, and some bacteria had not completely recovered even by Day 15, indicating that the reconstruction of dental plaque occurred gradually over time.

### The recovery of plaque flora was strongly correlated to the plaque index and bleeding index

**Fig. 4:**
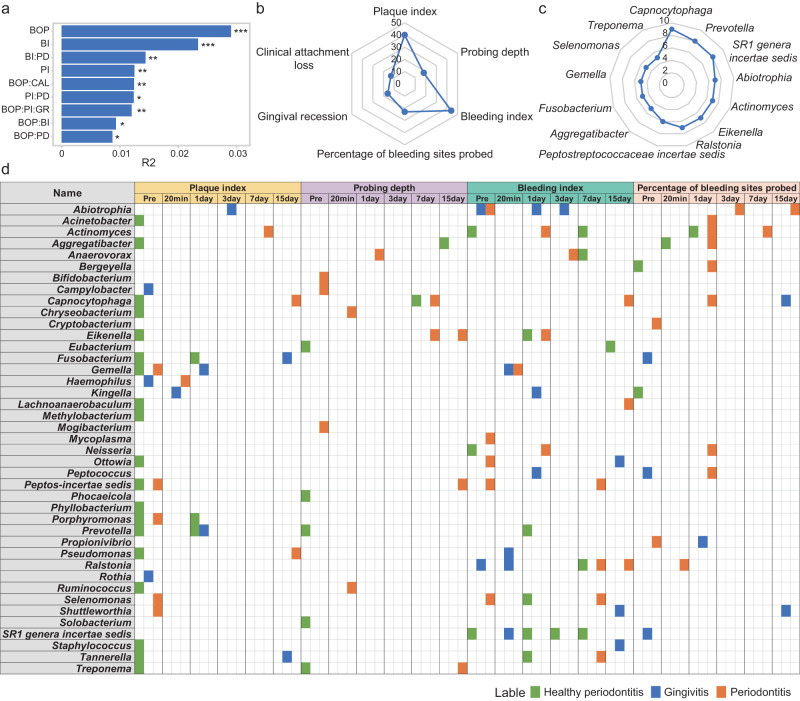
The correlation between clinical indicators and microorganisms. **a** Interaction between clinical indicators and bacteria associated with different periodontal statuses. BOP represents percentage of bleeding sites probed, BI represents bleeding index, PI represents plaque index, PD represents probing depth, GR represents gingival recession, CAL represented clinical attachment loss, PERMANOVA test was used. * for *P* < 0.05, ** for *P* < 0.01, *** for *P* < 0.001. **b** The number of bacteria associated with clinical indicators in different periodontal states at different times at the genus level. **c** The number of clinical indicators associated with bacteria at the genus level in different periodontal states at different times, greater than 5 (shown using Spearman correlation statistics). **d** The correlation between clinical indicators before scaling and plaque microbes at different time points before and after scaling in different periodontal states at the genus level. Pre represents pre-scaling, The green box represents healthy periodontitis, the blue box represents gingivitis, and the orange box represents periodontitis. *Peptos-incertae sedis* represents *Peptostreptococcaceae incertae sedis*. Using Spearman correlation statistics, and *P* < 0.05 was considered significant.

To quantify the relationship between plaque remodeling and the degree of periodontal health after scaling, we performed a reciprocal analysis of six clinical indices, including the percentage of bleeding site on probing, plaque index, bleeding index, probing depth, gingival recession and clinical attachment loss before scaling, and bacteria in different periodontal health states. We found that the percentage of bleeding sites on probing had the most significant effect on bacteria (Fig. 4a, *P* < 0.001, PERMANOVA test), followed by the bleeding index and plaque index. The bleeding index and probing depth were related to each other, indicating that these clinical indicators would not only affect periodontal health alone, but also cooperated with other indicators to affect periodontal health status.

We conducted correlation analysis between the six clinical indicators and all microorganisms before scaling. Initially, we evaluated whether the relative abundance obtained from 16S rRNA sequencing accurately reflected the true abundance of these microorganisms within the oral microbiome. We randomly selected 4 bacterial species and quantified using qPCR to assess their actual presence (Supplementary Fig. 8). The results demonstrated a strong correlation between the absolute copies and the relative abundance obtained through sequencing (Supplementary Fig. 9). A total of 41 bacterial genera were related to the six indicators of different healthy periodontal states (Supplementary Fig. 10). Furthermore, we calculated the correlation of each of the six clinical indicators with these genera at different time points after scaling (Fig. 4b–d and Supplementary Fig. 11). The results showed that the clinical indicators with the largest number of correlations with bacteria were bleeding index, plaque index and percentage of bleeding sites on probing (Fig. 4b), indicating that these indicators had a significant correlation with certain bacteria. The bacteria with the most clinical indicators at different time points in different periodontal states were *Capnocytophaga*, *Prevotella*, *SR1 genera incertae sedis* and *Abiotrophia* (Fig. 4c), suggesting that these bacteria might have a greater correlation with clinical indices.

Based on the changes in plaque microorganisms before and after scaling (Fig. 4d), it was observed that the indicators of plaque response were different for each period of plaque recovery in different periodontal health states. In the same periodontal health state, the bacteria associated with the indicators of periodontal status varied at different time points. Compared to healthy individuals and periodontitis patients, the bacterial genera in gingivitis patients were more affected by the plaque index during reconstruction after scaling. Unlike other clinical indices, the response of bacteria to the bleeding index was greater in all three states, and the bacteria of different states corresponded to the index at almost every time point, indicating that bleeding had a significant impact on the periodontal flora. The percentage of bleeding sites on probing, another indicator related to bleeding, increased as the severity of periodontal disease increased, along with the number of associated bacteria.

At the genus level, we found that five genera, such as *SR1 genera incertae sedis*, *Fusobacterium* and *Prevotella*, were only associated with periodontal health and gingivitis plaques in individuals. The *SR1 genera incertae sedis* before scaling and at Days 1, 3, and 7 after scaling were correlated with the bleeding index. In gingivitis plaques, the *SR1 genera incertae sedis* at 20 min after scaling was also correlated with the bleeding index, while before scaling, it was significantly correlated with the percentage of bleeding sites probed. Only six genera, including *Abiotrophia*, *Campylobacter* and *Haemophilus*, were associated with gingivitis and periodontitis plaques. *Abiotrophia* was correlated with plaque index at Day 3 and correlated with bleeding index before scaling and at Days 1 and 3 after scaling in gingivitis plaques. In periodontitis plaques, *Abiotrophia* at pre-scaling was associated with the bleeding index and the percentage of bleeding sites on probing at Days 3 and 15 after cleaning, suggesting that these bacteria might be key bacteria in the transition from gingivitis to health or periodontitis.

### The third day after plaque cleaning was a special period related to periodontal health status

**Fig. 5:**
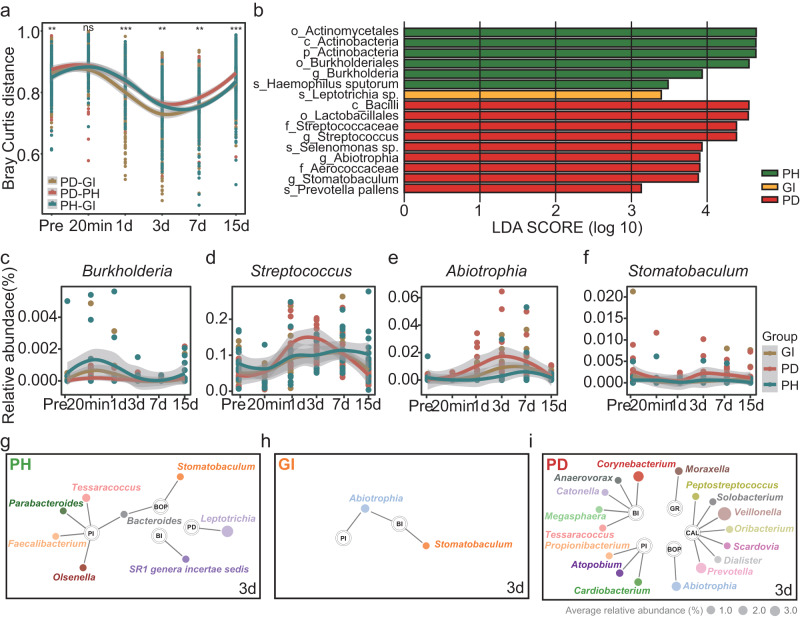
Differences in dental plaque and the dynamics of microbial abundance over time in different periodontal health states before and after scaling. **a** Bray‒Curtis distance among different periodontal health states at ASVs level. Green represents the Bray‒Curtis distance between the PH sample and the GI sample, red represents the Bray‒Curtis distance between the PH sample and the PD sample, and yellow represents the Bray‒Curtis distance between the GI sample and the PD sample. * represents *P* < 0.05, ** represents *P* < 0.01, and *** represents *P* < 0.001. **b** Differential bacteria in different periodontal health states at Day 3 after scaling at ASVs level. **c**–**f** Abundance of *Burkholderia*, *Streptococcus*, *Abiotrophia* and *Stomatobaculum* bacteria at the genus level. **g** The correlation of clinical indicators before scaling with bacteria at Day 3 after scaling in healthy periodontal plaques at the genus level. **h** The correlation of clinical indicators before scaling with bacteria at Day 3 after scaling in gingivitis plaques at the genus level. **i** The correlation of clinical indicators before scaling with bacteria at Day 3 after scaling in periodontitis plaques at the genus level. Spearman’s correlation tests were used. PH represents healthy periodontitis, GI represents gingivitis, and PD represents periodontitis.

In our earlier studies, we found that the third day after scaling was key period for the reconstruction of dental plaque biofilm^18^. Therefore, in this study, we focused on the dental plaque biofilm in different periodontal health conditions after scaling and found that the bacteria related to clinical indicators on the third day were the least quantity (Fig. 4d). This indicated that the third day was the least relevant to the indicators of periodontal status and could be an important day for controlling the course of plaque development. Furthermore, to determine the intervention period and intervention targets, we calculated the BC distance between the three groups to determine the similarity in different periodontal states. Based on the results, the flora of the groups was most similar at Days 3–7 after scaling. The distance between gingivitis and periodontitis plaques was closest at Day 3 (Fig. 5a, *P* = 0.0024, ANOVA test), suggesting that Day 3 after scaling was a special period in which possibly periodontitis-associated bacteria in the gingivitis group played a role.

Then, we analyzed the differences in dental plaque in volunteers with different periodontal health statuses at Day 3 after scaling. By linear discriminant analysis, we found 16 different differential genera in the three health states, of which gingivitis plaques had the least specific bacteria (only *Leptotrichia*) (Fig. 5b), indicating that the flora in this state was similar to that of periodontal health or periodontitis states. In the periodontally healthy periodontium, six bacteria, including Actinobacteria and Actinobacteriota, were the dominant bacteria; some of these bacteria were also dominant in the periodontium before scaling (Supplementary Fig. 12). In periodontitis plaques, nine bacteria such as *Abiotrophia* and *Prevotella pallens*, were the dominant bacteria, and it is possible that these bacteria played an important role in the formation of plaque in periodontitis states. Based on the bacteria in different periodontal health statuses on the third day, the bacteria clearly switched over time after scaling (Fig. 5c–f and Supplementary Fig. 13). Eleven marker taxa were also identified through taxonomic assignment based on the SILVA database (Supplementary Fig. 14). The dominant bacteria in periodontal health plaques switched greatly in periodontal healthy, and the dominant bacteria in periodontitis plaques switched greatly in periodontitis. Meanwhile, the trend of these bacteria in gingivitis was intermediate, indicating that perhaps these bacteria might play a unique role in the reconfiguration process. To revalidate the distribution of these genera across the different groups, we employed songbird, a computational tool capable of estimating differential abundance without the need for quantifying total microbial load or microorganism count, thereby minimizing the occurrence of false discoveries. Our findings further unveiled the associations of *Stomatobaculum* and *Streptococcus* exclusively with the periodontitis group. *Abiotrophia* exhibited associations with both the gingivitis and periodontitis groups (Supplementary Fig. 15).

Furthermore, we calculated the correlation between all genera in the different periodontal states on Day 3 and the clinical indicators before dental cleaning to identify bacteria that might have a potential impact on the recovery of periodontal health (Fig. 5g–i). We found that *Abiotrophia* was significantly associated with both the plaque index and bleeding index in gingivitis plaques, and it was also associated with the percentage of bleeding sites probed in periodontitis plaques. This result indicates that the discovery of the *Abiotrophia* at a specific time point might be important for the effective prevention and treatment of periodontal disease in the future.

## Discussion

Although there have been studies on the remodeling of the plaque biofilm in healthy individuals after removing plaque biofilm^19^ and in periodontitis patients after removing subgingival bacteria^20^, this was the longitudinal study on the microbial composition of suprgingival plaque and periodontal clinical indicators in different periodontal health states. We found that the changes in microbial communities in different periodontal states after supragingival scaling were not synchronized, and the more severe the periodontal damage was, the more delayed and switched the recovery of plaque flora after removal, reflective of a higher degree of plaque development in periodontitis. Understanding the ecological interpretation of stability of oral microbial communities over time in different periodontal health states is essential in utilizing these microbial communities to promote human health.

The 16S rRNA sequencing technique has been shown to rapidly classify, identify, and accurately quantify microorganisms in a community, and it has become the most commonly used method to study the microbiome independently of qPCR. This technology has been extensively used in the study of periodontal disease^21–23^. However, the technology is to annotate bacteria by comparing reference sequences with existing databases with high similarity, so bacteria may be inaccurately classified, and the ability of technology to obtain nearly “absolute” quantification by sequencing based methods is still controversial^24–26^. To reduce errors due to taxonomic assignment, we also used the SILVA database for taxonomic classification. Based on this database, we get similar results to those based on the RDP database (Fig. 2a–c and Supplementary Fig. 4). Further, based on different databases, we found that most marker bacteria found in different periodontal health states on the third day were overlapping (Fig. 5b and Supplementary Fig. 12), suggesting that these overlapping bacteria were more reliable. It should be noted that studies have suggested that the characteristic bacteria we found, such as *Burkholderia*^27^ and *Ralstonia*^28^, are easy to grow and have a high risk of waterborne genera and oral contaminants, although studies have also reported the presence of these bacteria in the oral cavity^29,30^. These disputes may be related to the molecular definition of the species, or to this particular population and their environment/habits, besides the possibility of contamination. Based on existing classification methods, we cannot rule out contamination or inaccurate classification of the reference sequences, suggesting that we need to be careful when using these bacteria as targets for some interventions or markers. In this study, a variety of combination methods were used to minimize the possible errors due to sequencing methods, including DEICODE, songbird, ANCOM-BC, and ALDEx2^31–34^, and relatively consistent results were obtained. Meanwhile, in order to verify the accuracy of the quantitative of 16S rRNA sequencing, the absolute copies of bacteria amplified by qPCR was significantly positively correlated with their relative abundance (Supplementary Figs. 8 and 9), which also proved the high reliability of the analysis results.

Although dental plaque biofilms contain both beneficial and harmful bacteria, their proportion shows a stable change trend with the maturation of dental plaque biofilms^35^, which depends on bacterial interactions and host-derived factors^36^. Previous studies have found that *Streptococci*, *Neisseria* and *Rothia* were the main early colonizers of tooth surfaces^37^, *Streptococci* expressed adhesins, especially α-amylase-binding protein A, antigen I/II, SspA/SspB and surface lectin, that could recognize receptors on acquired enamel membrane proteins to colonize the tooth surface. With the maturity of the plaque, the proportion of facultative and anaerobic filamentous bacteria, such as *Actinomyces*, *Corynebacterium*, *Fusobacterium* and *Veillonella*, gradually increased^19^. The results of our study showed that the order of completion of bacterial colonization was different in different periodontal health states. In periodontitis plaques, *Fusobacterium* recovered to its initial state at Day 3 after scaling, while *Tannerella* recovered to its initial state at Day 7 after scaling. These findings suggest that the periodontal health of the host should be considered in future studies of dental plaque colonization.

While traditional cognition studies have focused on changes in periodontitis-related pathogenic bacteria like *Porphyromonas gingivalis*, *Tannerella forsythia*, and *Treponema denticola*^38^, our study found that nontraditional pathogens of periodontitis were also important. These included *Capnocytophaga*, *SR1 genera incertae sedis* and *Abiotrophia*, which were found to have relationships with clinical indicators related to periodontal health status. We also discovered that *Abiotrophia* was significantly associated with bleeding in gingivitis and periodontitis plaques. Studies have shown that *Abiotrophia* is a resident of the human oral cavity, urogenital tract and intestinal tract, and its growth requires the support of vitamin B6 analogs. *Abiotrophia* is one of the causes of infective endocarditis, and can produce hydrogen sulfide, which affects the metabolism and ecology of dental plaque and is a virulence factor of periodontal disease^39^. The fibronectin binding ability of *Abiotrophia*, which is thought to be related to the pathogenicity of the organism, depends on the pH value. Bacteria can bind to immobilized fibronectin under appropriate pH culture conditions, and when the pH is higher than the appropriate pH, the binding capacity is greatly reduced or no binding occurs^40^. *Abiotrophia defectiva* was found to be susceptible to ampicillin, amoxicillin ceftriaxone, cefaclor, imipenem, ciprofloxacin, and vancomycin^41^. Collectively, these results suggest that *Abiotrophia* and other bacteria play an important role in determining the development direction of dental plaque biofilm development. Further intervention in *Abiotrophia* at this time point might be effective for the prevention and treatment of periodontal disease.

Furthermore, a previous study found that the three periodontal health states were dominated by 37 pathways, such as fatty acid biosynthesis, lipopolysaccharide biosynthesis, carbon fixation pathways, bacterial chemotaxis, and flagellar assembly. However, significant differences in bacterial interactions and functional profiles were observed between healthy and periodontitis plaques^15^. As the transformation from healthy to gingivitis states, full activation of 11 salivary cytokines and a synergetic decrease of plaque-derived betaine and *Rothia* spp. were reported. At this stage, the composition and functional characteristics of the dental plaque flora were highly similar to those of the periodontitis states^42^. This was found to be the key link between gingivitis, periodontitis and aging. These results suggest that the change in the microbiota during the shift from a healthy to periodontitis state was accompanied by changes in the structure and complexity of bacterial networks. Further functional studies on bacteria are needed to better understand the mechanisms underlying this shift.

Numerous studies have shown that the periodontal clinical indicators, serum markers and oral microorganisms were interrelated, for instance, the elevated serum lipopolysaccharide concentration in periodontitis patients indicated that the lipopolysaccharide from plaque could penetrate the gum or entered the circulation through the inflamed periodontal pocket opening^43^. Serum lipopolysaccharide concentration positively correlated with serum antibody response to major periodontal pathogens like *Actinobacillus actinomycetemcomitans* and *Porphyromonas gingivalis*^44^ as well as the number of probing bleeding and suppurative periodontal pockets^45^. Periodontitis increased levels of triglycerides and cholesterol in peripheral blood. *Fusobacterium nucleatum* was enriched in the mouth and liver and promotes glycolysis and lipogenesis of hepatocytes via the PI3K/Akt/mTOR signal^46^. Interleukin-17 levels increased in patients with periodontitis, and its production was related to the formation of bacterial community disorders^47^. Inhibition of interleukin-17 could significantly reverse the increased oral microbiome pathogenicity^48^, suggesting that interleukin-17 was a key mediator in this process. Additionally, 42 blood clinical markers have been associated with microbial diversity, such as fasting insulin, high-sensitivity C-reactive protein, alanine aminotransferase, low density lipoprotein and omega-3 fatty acids^49^. Omega-3 fatty acids supplementation reduced the probing depth of periodontal pockets in patients with periodontitis and increased the clinical attachment level^50^. It was speculated that periodontitis might be improved by improving the body’s serum concentration. Our study added new evidence to the association between periodontal clinical markers, serum markers, and oral microorganisms. They were effective inducers of inflammatory mediators in human peripheral blood, including interleukin-1β, interleukin-6, interleukin-8, tumor necrosis factor α, monocytechemoattractantprotein-1^51^, *Abiotrophia* bacteria might have a certain role in determining dental plaque biofilm development, but the exact mechanism of action remained unclear and needs further research.

Dietary type and content are closely related to the oral microbiota composition. Nutrients in the human diet, such as sugars, fats, and vitamins, are important for microorganisms and can alter the oral microbiota^52^. The Mediterranean diet was associated with a lower incidence of periodontitis and individuals who follow the Mediterranean diet had a lower percentage of bleeding sites on probing index and lower plaque indexes. Patients with severe periodontitis consumed more protein, fat, more carbohydrates, and alcohol than patients with no/mild periodontitis^53^. Saturated fatty acid and vitamin C intake were consistently associated with the microbial richness and diversity^54^. The metabolite 3-carboxy-4-methyl-5-propylfuranylic acid was a biomarker of fish intake, and higher plasma levels were associated with a lower risk of gingivitis and a 26% lower risk of severe periodontitis^55^, suggesting that a proper diet might improve periodontal health. Moreover, studies have found that probiotics could effectively reduce bleeding, plaque index and probing depth in the treatment of periodontitis^56^. Pasteurized *Akkermansia muciniphila* reduced periodontal and systemic inflammation induced by *Porphyromonas gingivalis*^57^, suggesting that the addition of probiotics might be another effective treatment option.

Gingival inflammation caused by plaque accumulation is considered a key risk factor for the development of periodontitis. Therefore, controlling gingival inflammation is crucial for the primary prevention of periodontitis^58^. This paper presents a study of the removal and remodeling of supragingival plaque in healthy individuals, gingivitis patients and periodontitis patients, which included an analysis of microbiological data under cross-sectional conditions. Ideally, a continuous longitudinal sample would be collected from a healthy periodontal state to a gingivitis state up to a periodontitis state. Obtaining such samples clinically could be difficult, but this was a worthwhile direction for clinical research.

The development of periodontal disease takes a very long time, ranging from several years to several decades, which greatly increases the difficulty of studying the plaque formation process associated with periodontitis. Our study found that changes in microbial communities in different periodontal states were not synchronized, and the more severe the periodontal damage, the more lagged and switched the recovery of the plaque flora after removal. Day 3 after plaque cleaning was a special period associated with the state of periodontal health, and *Abiotrophia* was significantly associated with clinical bleeding in gingivitis and periodontitis patients. Therefore, exploring the formation process of dental plaques in periodontal disease has important clinical significance for early detection, diagnosis, and intervention. This research could not only help understand the interaction between microorganisms from the viewpoint of biofilm formation but also enable clinicians to identify specific bacteria in a timely manner to prevent or delay the onset of chronic periodontitis.

## Methods

### Subject recruitment

Patients were recruited from the stomatology department of Wenzhou People’s Hospital. This study was approved by the ethics committee of the hospital (ethics number: KY-2022-257), and volunteers agreed and signed informed consent documents. Volunteers with different periodontal health statuses were recruited, and all enrolled subjects were of Han nationality and permanent residents of Wenzhou with at least 20 teeth, including at least one first molar left in the mouth. All subjects were non-vegetarians who consumed three normal meals per day. The patients were not pregnant or lactating, and they did not have any systemic, metabolic or oral diseases, including dental caries, diabetes, digestive diseases, Sjogren’s syndrome, hypertension, or heart disease. Patients also did not smoke or drink, take antibiotics or probiotics within six months, or receive oral therapy of any kind.

All subjects underwent oral clinical examination, and relevant clinical indicators were recorded. Periodontal health diagnoses were given based on the following conditions: no tooth probing depth greater than 3 mm, no attachment loss, and ≤10% of bleeding sites on probing. Gingivitis was diagnosed based on the following conditions: gingival tissue was congested and edematous, bright red in color, and soft and elastic, with no periodontal pocket formation or attachment loss; X-ray showed that the distance from the top of the crest of the alveolar bone to the osseous boundary of the enamel was less than or equal to 2 mm, PD > 4 mm without teeth, a percentage of bleeding sites >10% on probing, and no bone loss on imaging. Chronic periodontitis was diagnosed based on the following conditions: probing depth ≥ 4 mm, radiographs showed that distance from the top of the alveolar bone ridge to the enamel bone boundary was greater than or equal to 2 mm, gingival tissue was congested and edematous, with bleeding on probing, periodontal pocket formation, and attachment loss ≥3 mm. The diagnosis of periodontitis, gingivitis, and periodontal health was made by two or more medical professionals.

### Sample collection

All volunteers underwent oral clinical examination before dental cleaning, and relevant clinical indicators, including percentage of probing bleeding sites, plaque index, bleeding index, representative probing depth, gingival recession and clinical attachment loss, were recorded. Supragingival scaling was performed on patients in all three study groups, and all the plaque was removed from all teeth. Supragingival plaque samples were collected before scaling and at 20 min, Days 1, 3, 7, and 15 after scaling. After the volunteers’ mouths were rinsed with sterile saline, the first maxillary molar in the volunteers’ mouth was selected (the second maxillary molar was selected for those without the first molar). Their saliva was separated using sterile dry cotton balls, the tooth surface was gently dried with three guns, which is a chip blowing and drying device for dental treatment machines that performs the function of water injection, air injection and spray, and autoclave-sterilized periodontal curettage was used to scrape the gingival plaque at the buccal, lingual, proximal and distal axial angles of the sampled teeth. The samples were then suspended in a centrifuge tube containing 1 mL phosphate buffer and stored at −80 °C. Subjects were instructed not to take antibiotics for 15 days after teeth cleaning (and to inform the experimenter if there were any special circumstances), to use pasteurized teeth brushing (The procedure involves selecting a soft-bristled toothbrush and positioning it at a 45° angle towards the direction of the root tip, with maxillary teeth upwards and mandibular teeth downwards. Part of the bristles enter the gingival sulci, while others are spread on the gingival margin and extend into the adjacent space as far as possible, in accordance with the gingival-dental junction. The bristles are then made to vibrate horizontally in the front and back directions for a short distance, with gentle pressure, for 4–5 times. The toothbrush moves only about 1 mm during the vibration, and 2–3 teeth are brushed at a time before moving to the next set of teeth), and to avoid eating and brushing their teeth for 2 h prior to sample collection. All procedures were performed by periodontists with standard training.

### qPCR

qPCR assay was performed on QuantStudio 5384 qPCR System (Thermo Fisher Scientific, USA). Reaction mixtures contained 5 μl of 2 × TB Green II (Takara, Beijing), 0.2 μl of 50 × Rox Reference Dye (Takara, Beijing), 1 μl of Template, 3.6 μl of ddH_2_O and 0.2 μl of each 10 μM Forward/Reverse primers. The primers of *Abiotrophia*:^59^ forward (5′-GTGCTAGAAGTGGCTAG-3′) and reverse (5′-CGRTCAAATTGCATHCCTTC-3′), the primers of *Lautropia*:^60^ forward (5′-GTCCTTTTCGTTCCCGCC-3′) and reverse (5′-CAAGGCGACGATCTGTAGCTGG-3′), the primers of *Prevotella*:^61^ forward (5′- GAACCTTACCCGGGCTTGAA-3′) and reverse (5′-TGACGACAACCATGCAGCAC-3′), the primers of *Selenomonas*:^61^ forward (5′-CTTCGGATCGTAAAGCTCTG-3′) and reverse (5′-TAGTTAGCCGTGGCTTCCTC-3′), were used. The cycling conditions included initial denaturation of 30 s at 95 °C followed by 40 cycles of 95 °C for 5 s, 55 °C for 30 s and 72 °C for 30 s. No-template qPCR controls were performed using 1 μl ddH_2_O instead of DNA template. The reaction conditions for the dissociation stage are 95 °C for 15 s, 60 °C for 1 min and 99 °C for 15 s, 60 °C for 15 s. Each reaction had three replicates to collect CT values. The quantitative data were analyzed using QuantStudio Design and Analysis Software (Thermo Fisher Scientific, USA). According to the standardized curve, the average copy number of three replicates was the final result of each sample.

### 16S rRNA amplification sequencing

Total genomic DNA was extracted using the FastDNA® SPIN Kit for Soil (MP Biomedicals, Santa Ana, CA) according to the manufacturer’s instructions. The integrity of genomic DNA was assessed by agarose gel electrophoresis, and the concentration and purity of genomic DNA were measured using the Nanodrop 2000 Spectrophotometer (Thermo Fisher Scientific, USA) and Qubit3.0 Fluorometer (Life Technologies, USA). The V3-V4 hypervariable regions of the 16S rRNA gene were amplified with the primers 341 F (5′-CCTACGGGNGGCWGCAG-3′) and 805 R (5′-GACTACHVGGGTATCTAATCC-3′) and then sequenced with a 2*250 bp paired-end mode by Genesky Biotechnologies Inc. (Shanghai, China) using Illumina NovaSeq 6000 sequencer.

### Sequencing data processing and analysis

The raw read sequences were processed in QIIME2^62^. The adaptor and primer sequences were trimmed using the cutadapt plugin. DADA2 plugin was used for quality control and to identify amplicon sequence variants (ASVs)^63^. Taxonomic assignments of ASVs representative sequences were performed with confidence threshold 0.8 by a pre-trained Naive Bayes classifier, which was trained on the RDP Release version 11.5, RDP Taxonomy18.

All analyses were performed in R version 4.1.0 within RStudio version 1.4.1717, and figures were created using the ggplot and ggpubr packages. For independent comparisons, we considered *P* < 0.05 to be significant. However, for all analyses regarding multiple comparisons, we used the Benjamini‒Hochberg method to correct for multiple testing.

In brief, alpha diversity analysis was performed using Chao1, Shannon, Simpson, ACE, and Observed ASVs indicators. Beta diversity, using Bray-Curtis distances, and principal coordinate analysis were calculated based on standardized ASVs using the vegan package, and DECODE analysis was calculated based on ASVs using in qiime2 (2023.2). The intergroup differences in the bacterial community composition were analyzed at the ASVs level by the Wilcoxon test, and differences in communities were analyzed by the PERMANOVA test.

The bacteria that differed among different periodontal health states were identified using linear discriminant analysis based on the exclusion of low abundance bacteria (abundance greater than 5 and present in at least 10 samples) at the genus level, LDA score greater than 2.0 was considered significantly different, and using songbird, ALDEx2 and ANCOM-BC analysis at the genus level in qiime2 (2023.2)^31–34^. The pheatmap package was used to create the heatmap. Changes in bacteria over time in different periodontal health states were calculated by normalizing the bacteria at the genus level. The abundance of bacteria at different time points after scaling was compared with that before scaling, with an increased in red and a decrease indicated in blue. The results were analyzed using the Wilcoxon test. Altered bacteria in different periodontal health states on the third day after scaling were identified based on their ASVs level using linear discriminant analysis, and the abundance of bacteria at different time points in different states were plotted based on the previously identified bacteria.

The interactions of six clinical indices, including the percentage of probing bleeding sites, plaque index, bleeding index, probing depth, gingival recession, and clinical attachment loss, with bacteria at the genus level after screening were calculated by the PERMANOVA test based on stepwise regression analysis to select the best combination. Spearman correlation analysis was used to calculate the correlation between clinical indicators and bacteria at genus level at different time points before and after scaling. The number of bacteria associated with clinical indicators and the number of clinical indicators associated with bacteria at genus level were represented by radar plots. The genera of bacteria associated with clinical indicators at different periodontal health states before and at the third day after scaling were plotted separately on the network.

### Reporting summary

Further information on research design is available in the Nature Research Reporting Summary linked to this article.

### Supplementary information

Reporting Summary

Supplementary Figures and Tables

## Data Availability

The raw sequence data reported in this study have been deposited in the Genome Sequence Archive (http://bigd.big.ac.cn/gsa) in National Genomics Data Center, Beijing Institute of Genomics (China National Center for Bioinformation), Chinese Academy of Sciences under the accession code CRA009601.
