# Supplementary material for: The recovery of the microbial community after plaque removal depends on periodontal health status
Source: NPJ Biofilms Microbiomes. 2023 Oct 7;9:75. doi: 10.1038/s41522-023-00441-0 (PMC10560279; doi:10.1038/s41522-023-00441-0)
Supplement: Supplementary file 1 — Reporting Summary [file 41522_2023_441_MOESM1_ESM.pdf]

nature portfolio

Corresponding author(s): Professor Jinfeng Wang

Last updated by author(s): Jul 27, 2023

# Reporting Summary

Nature Portfolio wishes to improve the reproducibility of the work that we publish. This form provides structure for consistency and transparency in reporting. For further information on Nature Portfolio policies, see our Editorial Policies and the Editorial Policy Checklist.

## Statistics

For all statistical analyses, confirm that the following items are present in the figure legend, table legend, main text, or Methods section.

| n/a | Confirmed | |
|---|---|---|
| ☐ | ☒ | The exact sample size (*n*) for each experimental group/condition, given as a discrete number and unit of measurement |
| ☐ | ☒ | A statement on whether measurements were taken from distinct samples or whether the same sample was measured repeatedly |
| ☐ | ☒ | The statistical test(s) used AND whether they are one- or two-sided<br>*Only common tests should be described solely by name; describe more complex techniques in the Methods section.* |
| ☐ | ☒ | A description of all covariates tested |
| ☐ | ☒ | A description of any assumptions or corrections, such as tests of normality and adjustment for multiple comparisons |
| ☐ | ☒ | A full description of the statistical parameters including central tendency (e.g. means) or other basic estimates (e.g. regression coefficient) AND variation (e.g. standard deviation) or associated estimates of uncertainty (e.g. confidence intervals) |
| ☐ | ☒ | For null hypothesis testing, the test statistic (e.g. *F*, *t*, *r*) with confidence intervals, effect sizes, degrees of freedom and *P* value noted<br>*Give P values as exact values whenever suitable.* |
| ☒ | ☐ | For Bayesian analysis, information on the choice of priors and Markov chain Monte Carlo settings |
| ☒ | ☐ | For hierarchical and complex designs, identification of the appropriate level for tests and full reporting of outcomes |
| ☐ | ☒ | Estimates of effect sizes (e.g. Cohen's *d*, Pearson's *r*), indicating how they were calculated |

*Our web collection on statistics for biologists contains articles on many of the points above.*

## Software and code

Policy information about availability of computer code

| | |
|---|---|
| Data collection | No software was used. |
| Data analysis | The raw read sequences were processed in QIIME2. The adaptor and primer sequences were trimmed using the cutadapt plugin. DADA2 plugin was used for quality control and to identify amplicon sequence variants (ASVs). Taxonomic assignments of ASVs representative sequences were performed with confidence threshold 0.8 by a pre-trained Naive Bayes classifier, which was trained on the RDP Release version 11.5, RDP Taxonomy. All analyses were performed in R version 4.1.0 within RStudio version 1.4.1717, and figures were created using the ggplot and ggpubr packages. |

For manuscripts utilizing custom algorithms or software that are central to the research but not yet described in published literature, software must be made available to editors and reviewers. We strongly encourage code deposition in a community repository (e.g. GitHub). See the Nature Portfolio guidelines for submitting code & software for further information.

## Data

Policy information about availability of data

All manuscripts must include a data availability statement. This statement should provide the following information, where applicable:

- Accession codes, unique identifiers, or web links for publicly available datasets
- A description of any restrictions on data availability
- For clinical datasets or third party data, please ensure that the statement adheres to our policy

Provide your data availability statement here.

## Research involving human participants, their data, or biological material

Policy information about studies with human participants or human data. See also policy information about sex, gender (identity/presentation), and sexual orientation and race, ethnicity and racism.

| | |
|---|---|
| Reporting on sex and gender | Sex and/or gender was determined based on self-reporting. |
| Reporting on race, ethnicity, or other socially relevant groupings | All enrolled subjects were of Han nationality by self-reporting. |
| Population characteristics | Volunteers with different periodontal health statuses were recruited, with at least 20 teeth, including at least one first molar left in the mouth. All subjects were non-vegetarians who consumed three normal meals per day. The patients were not pregnant or lactating, and they did not have any systemic, metabolic or oral diseases, including dental caries, diabetes, digestive diseases, Sjogren's syndrome, hypertension, or heart disease. Patients also did not smoke or drink, take antibiotics or probiotics within six months, or receive oral therapy of any kind. |
| Recruitment | Patients were recruited from the stomatology department of Wenzhou People's Hospital. |
| Ethics oversight | the ethics committee of the Wenzhou People's Hospital |

Note that full information on the approval of the study protocol must also be provided in the manuscript.

# Field-specific reporting

Please select the one below that is the best fit for your research. If you are not sure, read the appropriate sections before making your selection.

☒ Life sciences ☐ Behavioural & social sciences ☐ Ecological, evolutionary & environmental sciences

For a reference copy of the document with all sections, see nature.com/documents/nr-reporting-summary-flat.pdf

# Life sciences study design

All studies must disclose on these points even when the disclosure is negative.

| | |
|---|---|
| Sample size | We recruited a total of 40 volunteers with varying periodontal health statuses for this study, including 13 with periodontal health, 15 with gingivitis and 12 with periodontitis. All volunteers underwent supragingival scaling, and we collected supragingival plaque samples at 6 time points before scaling and at 20 minutes, Day 1, Day 3, Day 7 and Day 15 after scaling. |
| Data exclusions | The V3-V4 region of the 16S rRNA gene was sequenced in each sample. Samples where DNA was not successfully extracted or amplification failed were excluded. Finally, 206 samples were successfully sequenced, yielding a total of 1,329,700 reads (2×250 bp), with an average of 64,549 121 reads per sample. |
| Replication | Plaque was collected at least 3 times at each site, and pooled together. DNA was extracted from the combined samples and the 16S rRNA gene was amplified, with each amplification containing a blank control using water as a template. |
| Randomization | All volunteers underwent oral clinical examination before dental cleaning, and relevant clinical indicators, including percentage of probing bleeding sites, plaque index, bleeding index, representative probing depth, gingival recession and clinical attachment loss, were recorded. |
| Blinding | The doctors who collected the sample were blinded to group allocation during data analysis. |

# Reporting for specific materials, systems and methods

We require information from authors about some types of materials, experimental systems and methods used in many studies. Here, indicate whether each material, system or method listed is relevant to your study. If you are not sure if a list item applies to your research, read the appropriate section before selecting a response.

## Materials & experimental systems

| n/a | Involved in the study |
|---|---|
| ☒ | ☐ Antibodies |
| ☒ | ☐ Eukaryotic cell lines |
| ☒ | ☐ Palaeontology and archaeology |
| ☒ | ☐ Animals and other organisms |
| ☒ | ☐ Clinical data |
| ☒ | ☐ Dual use research of concern |
| ☒ | ☐ Plants |

## Methods

| n/a | Involved in the study |
|---|---|
| ☒ | ☐ ChIP-seq |
| ☒ | ☐ Flow cytometry |
| ☒ | ☐ MRI-based neuroimaging |
