# Supplementary material for: The recovery of the microbial community after plaque removal depends on periodontal health status
Source: NPJ Biofilms Microbiomes. 2023 Oct 7;9:75. doi: 10.1038/s41522-023-00441-0 (PMC10560279; doi:10.1038/s41522-023-00441-0)
Supplement: Supplementary file 2 — Supplementary Figures and Tables [file 41522_2023_441_MOESM2_ESM.pdf]

Supplementary Figures and Tables

Category	Variables	PH (n=13)	GI (n=15)	PD (n=12)	*P* value
General information	Ethnic Han (%)	100	100	100	-
	Living area (Wenzhou, China) (%)	100	100	100	-
	Gender(Female)(%)	92.3	86.7	75	ns
	Smoking (%)	0	0	0	-
Medications	Probiotic supplement (%)	0	0	0	-
	Use insulin (%)	0	0	0	-
History of diseases	Diabetes	0	0	0	-
	Sjogren's syndrome	0	0	0	-
	Stomach disease	0	0	0	-
	Family history of periodontal disease	0.076923077	0.2	0.083333333	ns
Dietary habits	Vegetarianism (%)	0	0	0	-
	Three meals per day (times/day)	100	100	100	-
	Tea (%)	15.38	20	8.33	
	Carbonated drinks (%)	0	0	0	-
Dental care habits	Daily frequency of brushing	2.231±0.4385	2±0	1.833±0.3892	*
	Brushig time (min/per)	2.154±0.6887	1.333±0.7237	1.833±0.8348	*
Clinical indexes	BOP (%)	3.846±1.068	15.67±2.498	58.08±4.231	****
	PI (%)	0.7692±0.4385	1.6±0.5071	2.333±0.4924	****
	BI (%)	0.3846±0.5064	2.4±0.6325	3.583±0.5149	****
	PD (mm)	2.077±0.4003	2.9±0.5732	4.542±0.6557	****
	GR (mm)	0	0	0.7083±0.4981	****
	CAL (mm)	0	0	5.25±0.6571	****

Supplementary Table 1. Clinical information of the patient

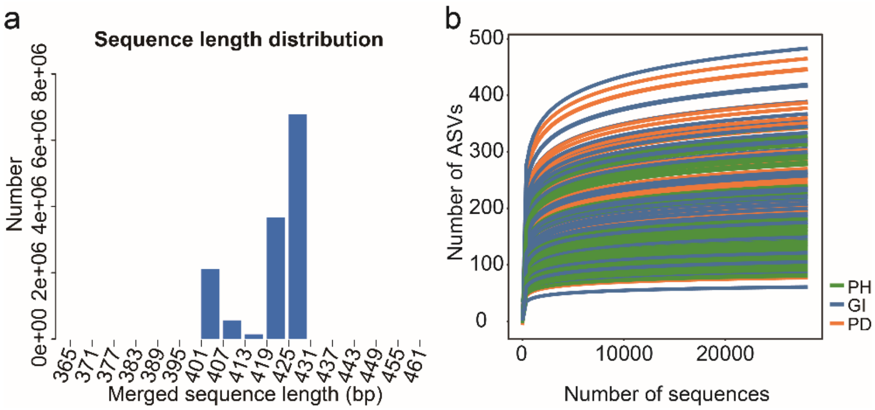

Supplementary Fig. 1 Summary of sequencing data. a. Length distribution of the merged paired-end sequences. b. Rarefaction curves of sequencing data.

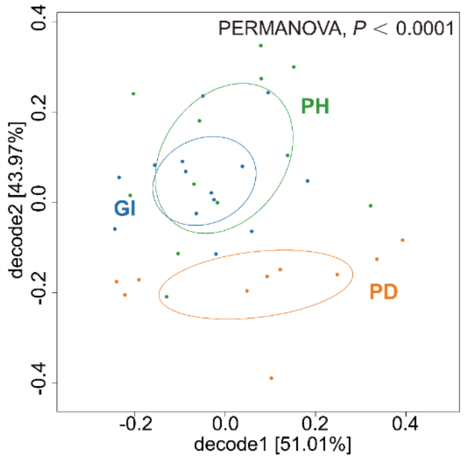

Supplementary Fig. 2 DECODE analysis results of different periodontal states at the ASV level, using PERMANOVA test.

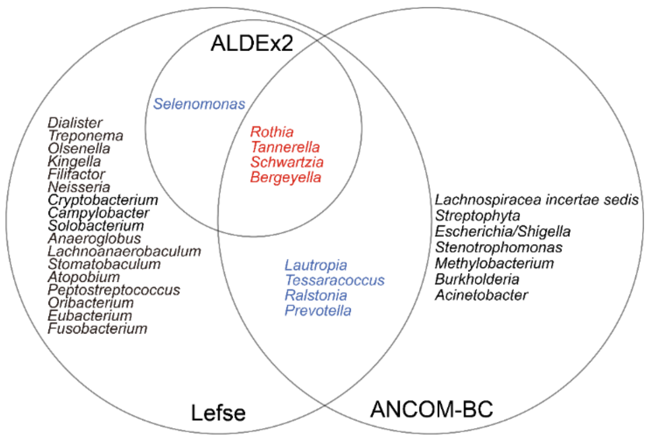

Supplementary Fig. 3 Differential bacteria in different periodontal states identified by LEfSe analysis, ALDEx2 analysis and ANCOM-BC analysis at the genus level.

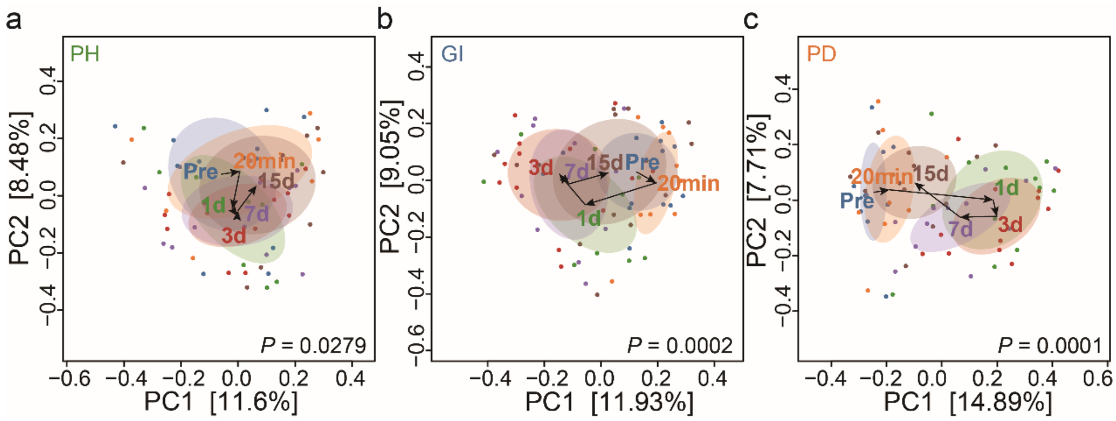

Supplementary Fig. 4 Changes in the microbial of dental plaque under different periodontal health conditions after scaling. a. Principal coordinates analysis for different time points before and after scaling in periodontal health samples at the ASVs level. b. Principal coordinates analysis for different time points before and after scaling in gingivitis samples at the ASVs level. c. Principal coordinates analysis for different time points before and after scaling in periodontitis samples at the ASVs level. Each point in the figure represents a sample, and each color represents a time point. The starting point of each arrow in the figure is the center of the circle for the group, and the direction of the arrow is a path, which is set according to the gradient direction of the continuous time after tooth cleaning (the order is pre-20min-1d-3d-7d-15d). Pre represents pre-scaling, PH represents healthy periodontitis, GI represents gingivitis, and PD represents periodontitis. Taxonomic assignments of ASV representative sequences were performed with confidence threshold 0.8 by a pre-trained Naive Bayes classifier which was trained on the SILVA (version 138.1).

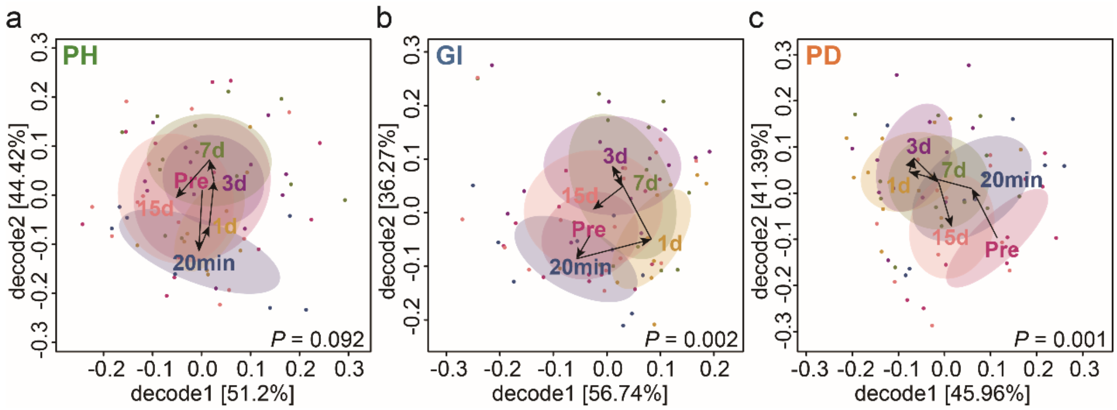

Supplementary Fig. 5 Changes in the microbial diversity of dental plaque under different periodontal health conditions after scaling. a. DECODE analysis for different time points before and after scaling in periodontal health samples at the ASV level. b. DECODE analysis for different time points before and after scaling in gingivitis samples at the ASV level. c. DECODE analysis for different time points before and after scaling in periodontitis samples at the ASV level. Each point in the figure represents a sample, and each color represents a time point. The starting point of each arrow in the figure is the center of the circle for the group, and the direction of the arrow is a path, which is set according to the gradient direction of the continuous time after tooth cleaning (the order is pre-20min-1d-3d-7d-15d). Pre represents pre-scaling, PH represents healthy periodontitis, GI represents gingivitis, and PD represents periodontitis.

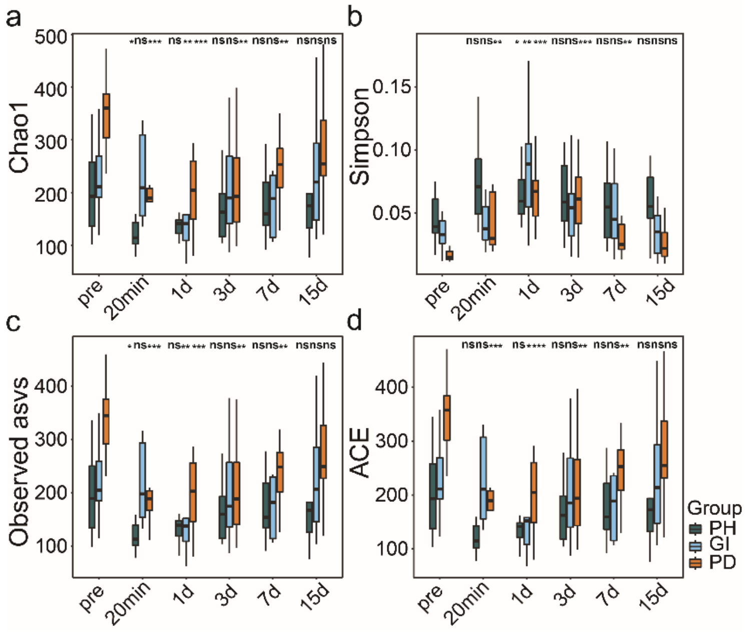

Supplementary Fig. 6 Alpha diversity of supragingival plaque in different periodontal health states. a. Chao1 index of different periodontal health states. b. Simpson index of different periodontal health status. c. Observed ASVs index of different periodontal health states. d. ACE index of different periodontal health status. Box plots showed center line as median, box limits as upper and lower quartiles, whiskers as 1.5 × interquartile range. Pre represents pre-scaling, PH represents healthy periodontitis, GI represents gingivitis, and PD represents periodontitis. * represents $P < 0.05$, ** represents $P < 0.01$, *** represents $P < 0.001$.

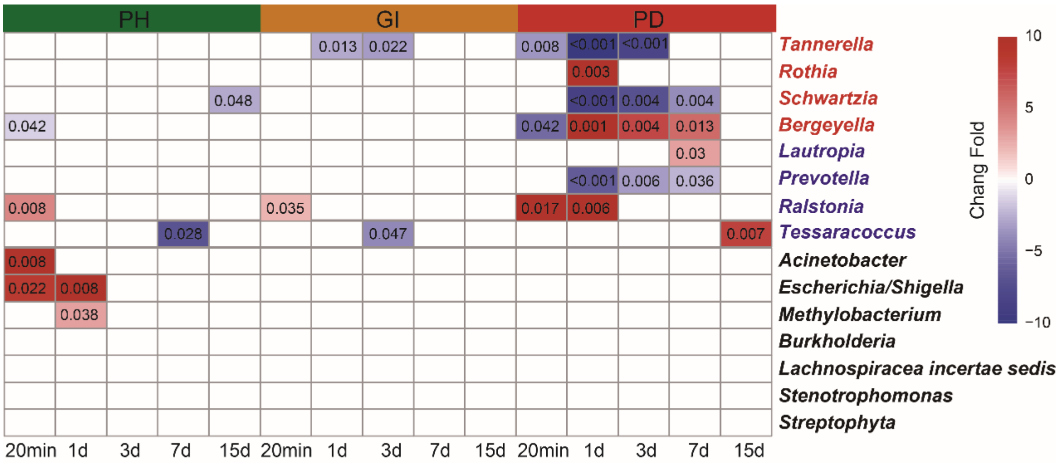

Supplementary Fig. 7 Changes in differential bacteria by ANCOM-BC analysis at different time points after scaling. Significantly different genera at each time point compared to before scaling at the genus level. Red shadows represent increased genera, while blue shadows represent decreased genera. The numbers in the box show *P-values* (Wilcoxon test). The red fonts represent the bacteria with significant differences after LEfSe analysis, ALDEx2, and ANCOM-BC analysis at the genus level. The blue fonts represent the bacteria with significant differences after LEfSe analysis and ANCOM-BC analysis at the genus level. PH represents healthy periodontitis, GI represents gingivitis, and PD represents periodontitis.

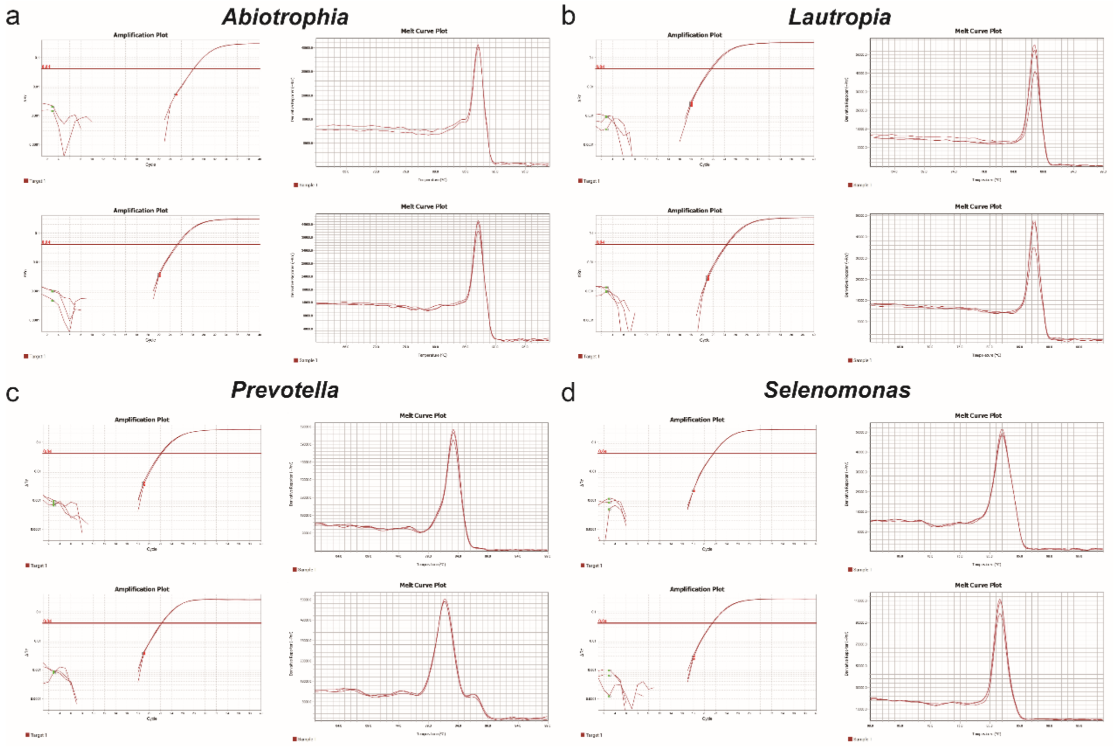

Supplementary Fig. 8 Amplification curve and melting curve of qPCR. a. The amplification curve and melting curve of *Abiotrophia* (partial data). b. The amplification curve and melting curve of *Lautropia* (partial data). c. The amplification curve and melting curve of *Prevotella* (partial data). d. The amplification curve and melting curve of *Selenomonas* (partial data).

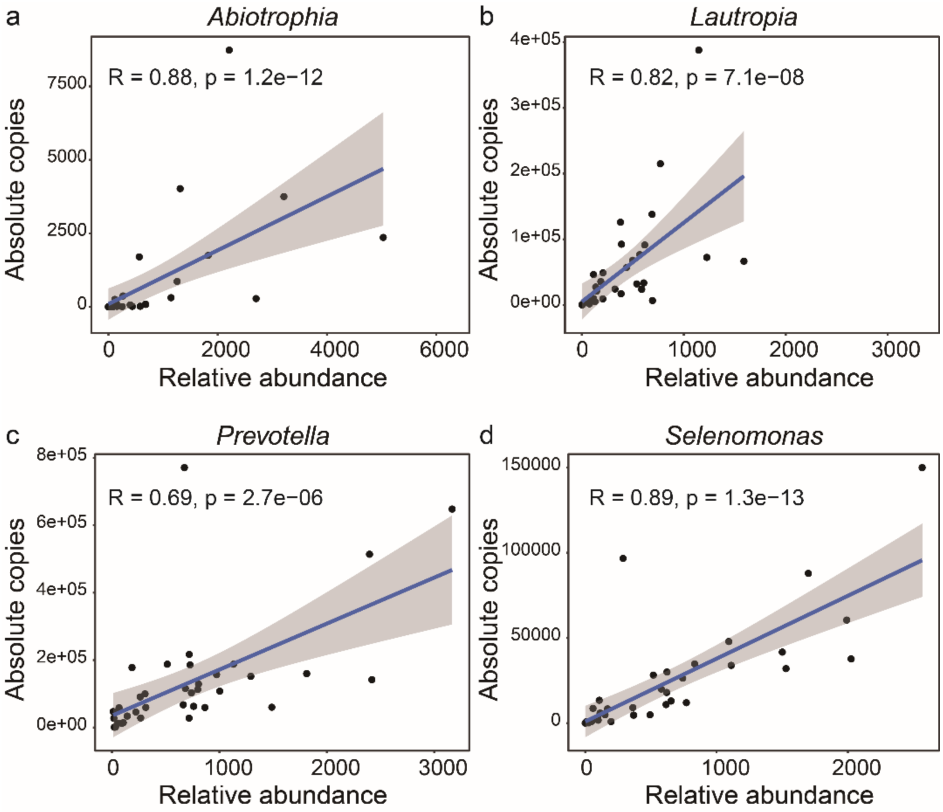

Supplementary Fig. 9 Correlation between relative abundance and absolute copies (N=54). a. Correlation between relative abundance and absolute copies of *Abiotrophia*. b. Correlation between relative abundance and absolute copies of *Lautropia*. c. Correlation between relative abundance and absolute copies of *Prevotella*. d. Correlation between relative abundance and absolute copies of *Selenomonas*. Spearman test was used.

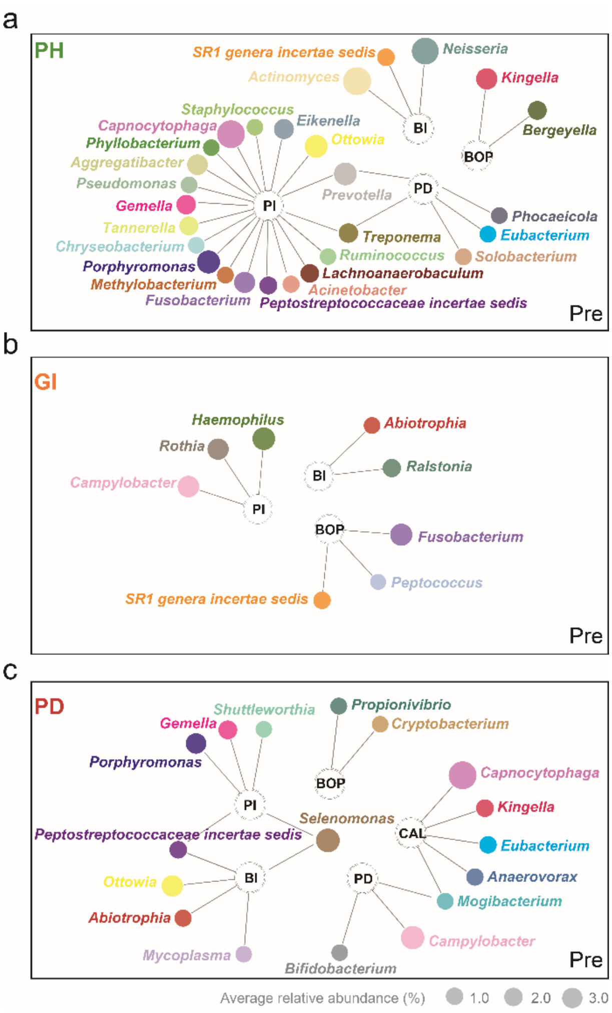

Supplementary Fig. 10 Correlation between clinical indicators and microorganisms before scaling. a. Correlation of clinical indicators with bacteria before scaling in healthy periodontal. b. Correlation of clinical indicators with bacteria before scaling in gingivitis. c. The correlation of clinical indicators with bacteria before scaling in periodontitis. Spearman's correlation tests were used. PH represents healthy periodontitis, GI represents gingivitis, and PD represents periodontitis.

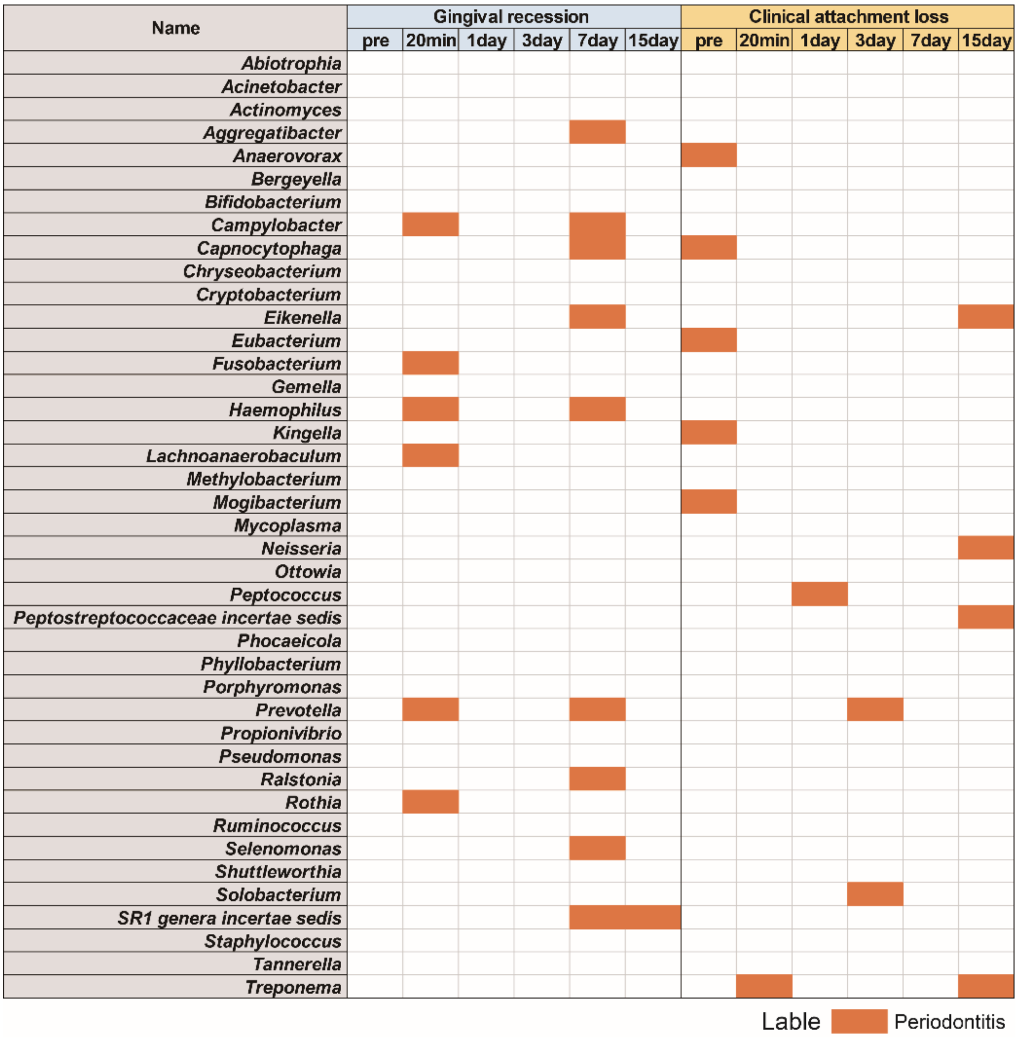

Supplementary Fig. 11 Correlation of clinical indicators with bacteria in periodontitis. Pre represents pre-scaling, and the orange box represents periodontitis.

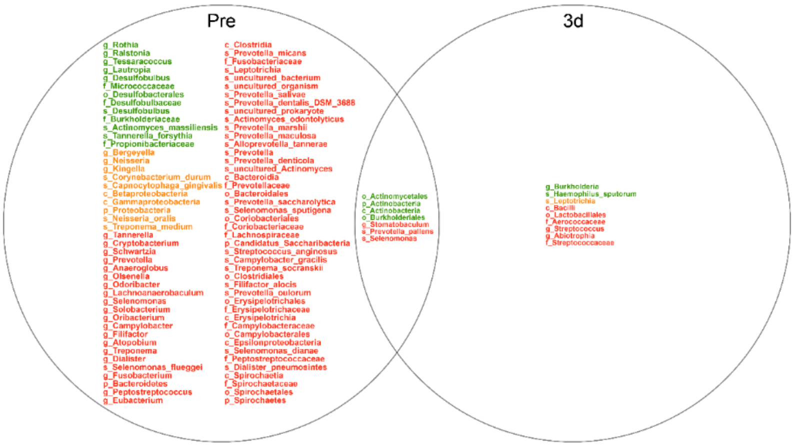

Supplementary Fig. 12 The differential bacteria before cleaning and Day 3 after cleaning identified by LEfSe analysis. Green represents the dominant bacteria of healthy periodontium, orange represents the dominant bacteria of gingivitis and red represents the dominant bacteria of periodontitis.

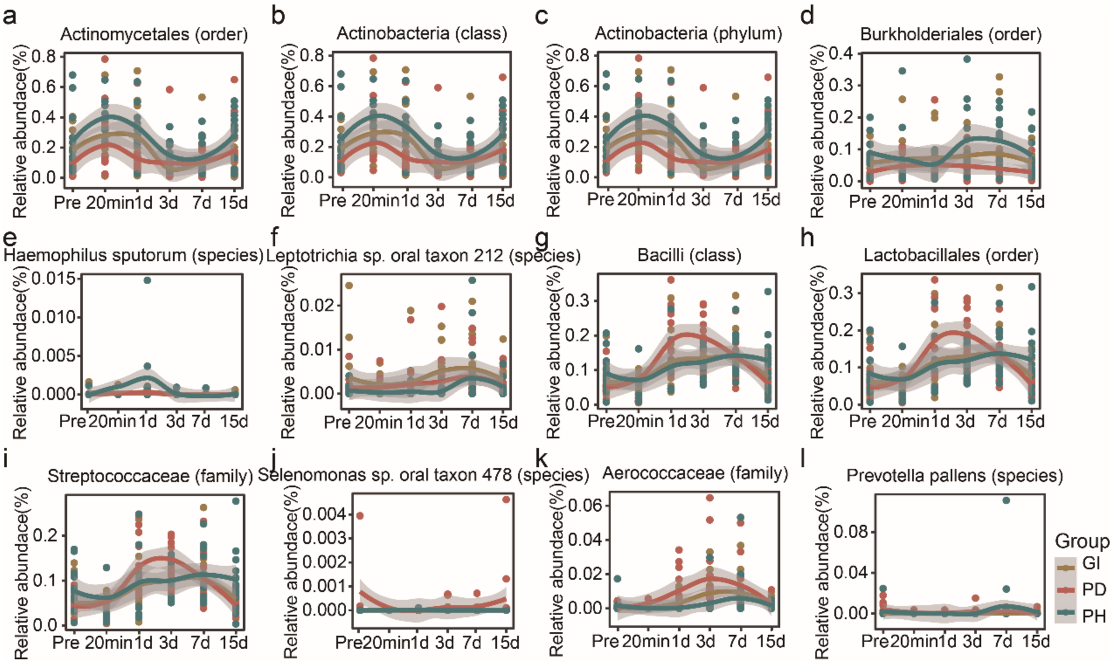

Supplementary Fig. 13 Dynamics of microbial abundance over time in different periodontal health states before and after scaling. a. The abundance of Actinobacteria at different time points at order level. b. The abundance of Actinobacteria at different time points at class level. c. The abundance of Actinomycetales at different time points at phylum level. d. The abundance of Burkholderiales at different time points at order level. e. The abundance of Haemophilus sputorum at different time points at species level. f. The abundance of Leptotrichia sp. oral taxon 212 at different time points at species level. g. The abundance of Bacilli at different time points at class level. h. The abundance of Lactobacillales at different time points at order level. i. The abundance of Streptococcaceae at different time points at family level. j. The abundance of Selenomonas sp. oral taxon 478 at different time points at species level. k. The abundance of Aerococcaceae at different time points at family level. l. The abundance of Prevotella pallens at different time points at species level. PH represents healthy periodontitis, GI represents gingivitis, and PD represents periodontitis.

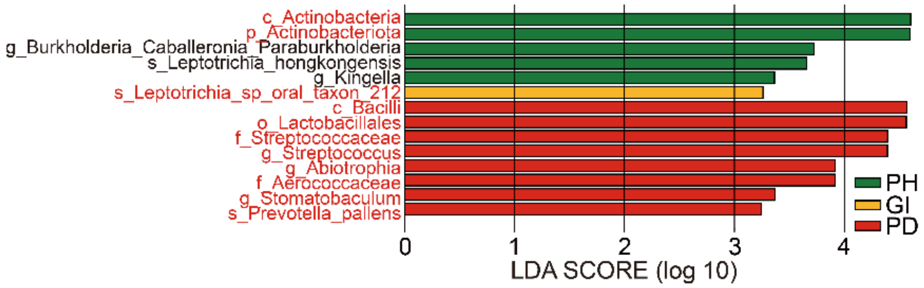

Supplementary Fig. 14 Differential bacteria in different periodontal health states at Day 3 after scaling at ASVs level. The red font represents the same marker bacteria based on the RDP database and SILVA database. Taxonomic assignments of ASV representative sequences were performed with confidence threshold 0.8 by a pre-trained Naive Bayes classifier which was trained on the SILVA (version 138.1).

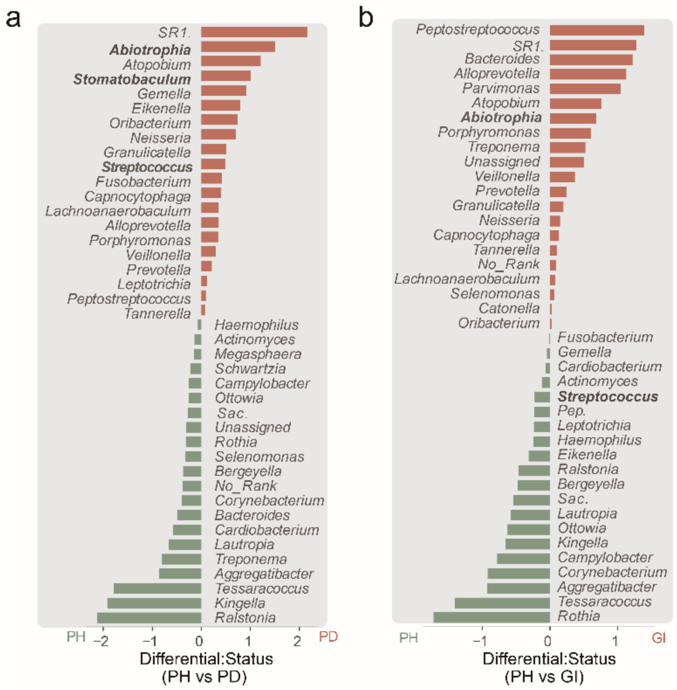

Supplementary Fig. 15 Songbird feature rankings of bacteria among different groups on the third day after scaling. a. Differential rankings of taxa associated with periodontitis and periodontal health. b. Differential rankings of taxa associated with gingivitis and periodontal health. *SR1*. represents *SR1 genera incertae sedis*, *Sac*. represents *Saccharibacteria genera incertae sedis*, *Pep*. represents *Peptostreptococcaceae incertae sedis*.
